# Psychophysical well-being and physical activity of Polish doctors

**DOI:** 10.3389/fpubh.2025.1608135

**Published:** 2025-07-02

**Authors:** Sławomir Wojczyk, Józefa Dąbek, Julia Bijoch, Magdalena Szynal

**Affiliations:** ^1^Department of Vascular and General Surgery, Provincial Specialist Hospital No. 4, Bytom, Poland; ^2^Department of Cardiology, Faculty of Health Sciences in Katowice, Medical University of Silesia in Katowice, Katowice, Poland; ^3^Collegium Medicum, Faculty of Medicine, WSB University, Dąbrowa Górnicza, Poland

**Keywords:** psychophysical well-being, physical activity, Polish physicians, mental well-being, physical well-being

## Abstract

**Background:**

Psychophysical well-being is a multidimensional concept involving positive emotions, life satisfaction, good health, and meaningful social relationships, essential for overall happiness and life success. Maintaining this well-being relies heavily on a healthy lifestyle, which significantly reduce health risks and improve quality of life. For doctors, whose work involves high mental and physical demands, psychophysical well-being is crucial, as stress and burnout can impair their health and the quality of patient care. The aim of the study was to assess the psychophysical well-being of Polish doctors and to analyzed physical activity of Polish doctors related to their psychophysical well-being.

**Methods:**

The study involved 832 (100%) physicians from hospitals located in the Silesian Voivodeship. To assess psychophysical well-being, with the authors’ consent, the theoretical D scale of the Psychosocial Working Conditions Questionnaire was used to measure the perceived level of well-being and 2 factors (empirical scales D1 and D2) regarding physical and mental well-being (together described as psychophysical well-being). Physical activity was assessed using the shortened International Physical Activity Questionnaire (IPAQ).

**Results:**

About 20% had a low level of psychophysical well-being, especially physical well-being. Men were characterized by significantly higher psychophysical well-being (*p* < 0.001), physical well-being (*p* < 0.001) and mental well-being (*p* < 0.001) than women, similarly to older doctors (psychophysical well-being *p* = 0.02, mental well-being *p* < 0.001) and those working in surgical wards (psychophysical well-being *p* < 0.01, mental well-being *p* < 0.01). Less than half of the doctors declared regular physical activity, mainly of low intensity, with women more often having a low level of activity (*p* < 0.001). No significant correlation was found between the level of psychophysical well-being and physical activity.

**Conclusion:**

The psychophysical well-being of Polish doctors was unsatisfactory, especially among women, younger doctors, those with less work experience, and those in non-surgical departments, and it was not related to their physical activity. The physical activity levels were average, with lower activity observed in female doctors and those not working on duty. There is a need to improve doctors’ physical activity habits and working conditions through educational and support programs.

## Introduction

The concept of psychophysical well-being is defined as a set of experiences consisting of experiencing positive emotions, lack of bad moods and a high level of satisfaction

with life. The aforementioned concept is also associated with a sense of optimism that affects health, well-being and life successes. Contemporary research emphasizes that psychophysical well-being is a multidimensional construct, encompassing both mental and physical aspects of human functioning. It includes positive emotions, life satisfaction, a sense of meaning, as well as good social relationships and physical health (1, 2). According to Carol Ryff, well-being is not only the absence of mental illness, but also the presence of positive qualities such as autonomy, mastery over the environment, personal growth, positive relationships with others, purpose in life, and self-acceptance (3). In literature psychological well-being is often identified with happiness, satisfaction or quality of life. The most popular definition of happiness in psychology is describing one’s life as close to ideal, the feeling that one has received everything one wanted from life and liking one’s life and not feeling the need to change. The subjective feeling of happiness can therefore be identified with psychophysical well-being (4).

As is well known, an important element necessary for maintaining happiness is full health. According to the World Health Organization (WHO), health is not only the absence of disease, but also the feeling of complete well-being, both mental and physical (5). Already in the 16th century, the Polish poet Jan Kochanowski wrote in his epigram “Noble health, nobody will know how you taste, until you spoil” (6). It means that a person appreciates health only when he loses it. Studies already published also prove that people enjoying good health declare a better quality of life (7).

In line with the proverb that “prevention is better than cure” - one strategy for maintaining health is to stop diseases from developing by encouraging a healthy lifestyle and avoiding risky behaviors. According to Lalonde’s health fields, lifestyle determines health by as much as 50%, while the environment and genetic load are only 20%, and medical care - only 10% (8). A healthy lifestyle includes, among others: proper nutrition, avoidance of stimulants (e.g., refraining from smoking), limitation of alcohol consumption, and regular, systematic physical activity. Physical activity contributes to improving cardiovascular efficiency, lowering blood pressure, increasing cardiac stroke volume, enhancing vascular elasticity, and reducing the risk of atherosclerosis and its complications. Moreover, it decreases the risk of stroke, enhances metabolic processes, supports the treatment of obesity and overweight, alleviates stress, improves cognitive functioning and logical reasoning, and strengthens concentration and memory (9–13). Research shows that regular physical activity promotes better quality and even prolongation of life (12, 14).

According to WHO recommendations, adults and healthy people should undertake moderate (150–300 min/week) or intense (75–150 min/week) exercise or an equivalent combination of moderate and intense exercise (15).

Especially physicians, have high self-awareness of the high value of leading a healthy lifestyle, including regular physical activity, as well as its importance in health promotion.

Taking up health-risk behaviors, such as: little or no physical activity, smoking, alcohol abuse, and exposure to chronic stress and lack of rest, are associated with the risk of developing circulatory system diseases. These diseases have been one of the main causes of disability, prolonged rehabilitation and related absence from work for years. Above all, remain the most common cause of death in highly developed countries. Physicians should be people who not only maintain healthy habits, but also promote them with their attitude, educating patients in this area.

In addition, the psychophysical well-being of doctors is a fundamental element of the functioning of an effective healthcare system. Doctors, as medical professionals, are exposed to exceptionally high mental and physical burdens, which can lead to serious health consequences and a decrease in the quality of patient care. The mental and physical condition of a doctor translates directly into the effectiveness of medical work. Occupational stress, chronic fatigue or burnout reduce the ability to concentrate, negatively affect clinical decision-making and empathy toward the patient. Another common social problem is burnout among doctors (16, 17). Locally, Poland has been struggling with a shortage of doctors for years, especially in some specializations and in smaller towns. The result is the need to work overtime and a lot of physical and mental strain.

The aim of the study was to assess the psychophysical well-being of Polish doctors and to analyzed physical activity of Polish doctors related to their psychophysical well-being.

## Materials and methods

The study involved 832 (100%) physicians from hospitals located in the Silesian Voivodeship. There are about sixteen thousand registered doctors in the mentioned voivodeship, of which about fifteen thousand are actively working. The average age of the respondents was 39.09 ± 10.67 years. The inclusion criteria for the study included: expressing informed and voluntary consent to participate in the study, having full rights to practice as a physician, and working in the profession for at least 1 year. The sample size was defined based on the number of responses received.

The study was approved by the Bioethics Committee of the Medical University of Silesia in Katowice (resolution no. PCN/0022/KB/287/19 of December 16, 2019). The study used original questionnaires characterizing the respondents (demographic data, years of employment, type of department, etc.) and Polish versions of standardized questionnaires. The questionnaires were given to the study participants in paper form, and the so-called snowball method was used to distribute them. Data collection lasted from September 2020 to September 2021.

To assess psychophysical well-being, with the authors’ consent, the theoretical D scale of the Psychosocial Working Conditions Questionnaire was used to measure the perceived level of well-being and 2 factors (empirical scales D1 and D2) regarding physical and mental well-being (together described as psychophysical well-being). The primary question of the theoretical scale (D) is “How do you feel?” The D1 scale consists of a general assessment of physical health and stress and the occurrence of somatic symptoms such as headaches, stomach and heart problems. Factors related to mental well-being (D2) focus on the assessment of negative emotional states, satisfaction with life and work, and self-confidence. High scores indicate a high level of well-being. The questionnaire is provided with standards developed for 8 professional groups, including nurses (15). These empirical scales D1 and D2 make up the theoretical scale D. The D scale has good internal consistency (Cronbach’s alpha above 0.7). Validity is confirmed by correlations with other indicators of stress, health, and job satisfaction (15).

Physical activity was assessed using the shortened International Physical Activity Questionnaire (IPAQ). It contains 7 questions regarding all types of physical activity related to daily life, work and leisure. Information is collected on time spent sitting, walking and time devoted to physical activity: vigorous and moderate. The results were classified and the subjects were divided according to the intensity of physical activity: low physical activity (less than 600 MET minutes/week) and moderate physical activity (between 600 and 1,500 MET minutes/week or 600 and 3,000 MET minutes/week depending on the number of days, intensity and time of physical activity) and high physical activity (over 1,500 MET minutes/week but at least 3 days a week with vigorous effort or 7 or more days of any combination of exercise exceeding 3,000 MET minutes/week) (18). MET stands for Metabolic Equivalent of Task. This is an indicator of the intensity of physical activity, defined as the ratio of metabolic rate during a given activity to metabolic rate at rest. It is conventionally assumed that 1 MET corresponds to oxygen consumption of 3.5 mL O₂ per kilogram of body weight per minute, which is equivalent to the resting metabolic rate of an average adult (19). IPAQ is a tool with moderate validity and good reliability in assessing physical activity in adult populations. Its adaptation to Polish was carried out in accordance with international standards (Cronbach’s alpha 0.75–0.80) (18).

Statistical analysis was performed using Statistica 13.3 software (Statsoft, Poland). Qualitative data were presented taking into account the number and percentages in relation to the entire group, while quantitative data were presented taking into account descriptive statistics, i.e.: mean, standard deviation and median. The aforementioned data were analyzed using the Shapiro–Wilk test to assess the occurrence of normal distribution (20). Due to the deviation of the described data from the normal distribution, nonparametric tests were used. The Mann–Whitney *U* test was used for comparisons between two groups, and the Kruskal-Wallis test was used between more than two groups (21, 22). Correlation analyses were performed using the Spearman test (23). Statistical significance was set at *p* < 0.05. There were no missing data in our study.

## Results

Table 1 presents the general characteristics of the studied group of physicians.

**Table 1:** Characteristics of the study group of doctors.

Variables		Number (%)
Sex	Women	510 (61.30%)
	Men	322 (38.70%)
Age [years]	25–30	205 (24.64%)
	31–40	312 (37.50%)
	41–50	176 (21.15%)
	51–60	101 (12.14%)
	>60	38 (4.57%)
Marital status	Married	552 (66.35%)
	Divorced	41 (4.93%)
	Single/bachelor	227 (27.28%)
	Widow/Widower	12 (1.44%)
Having offspring	Yes	516 (62.02%)
	No	316 (37.98%)
Specialization	Treatment	274 (32.93%)
	Non-surgical	558 (67.07%)
Working on duty	Yes	660 (79.33%)
	No	172 (20.67%)
Average working time on duty [hours]	I’m not on duty	164 (19.71%)
	≤12	96 (11.54%)
	13–24	430 (51.68%)
	25–48	120 (14.42%)
	>48	22 (2.64%)

The majority of the examined group of doctors were women (510; 61.30%), and the majority were between 31 and 40 years old (312; 37.50%). The vast majority (67.07%) of the examined performed non-surgical specialization, and less than 1/5 of the group (164; 19.71%) did not work on duty. The largest group among the examined doctors were doctors working in the profession from 1 to 10 years.

**Figure 1:**
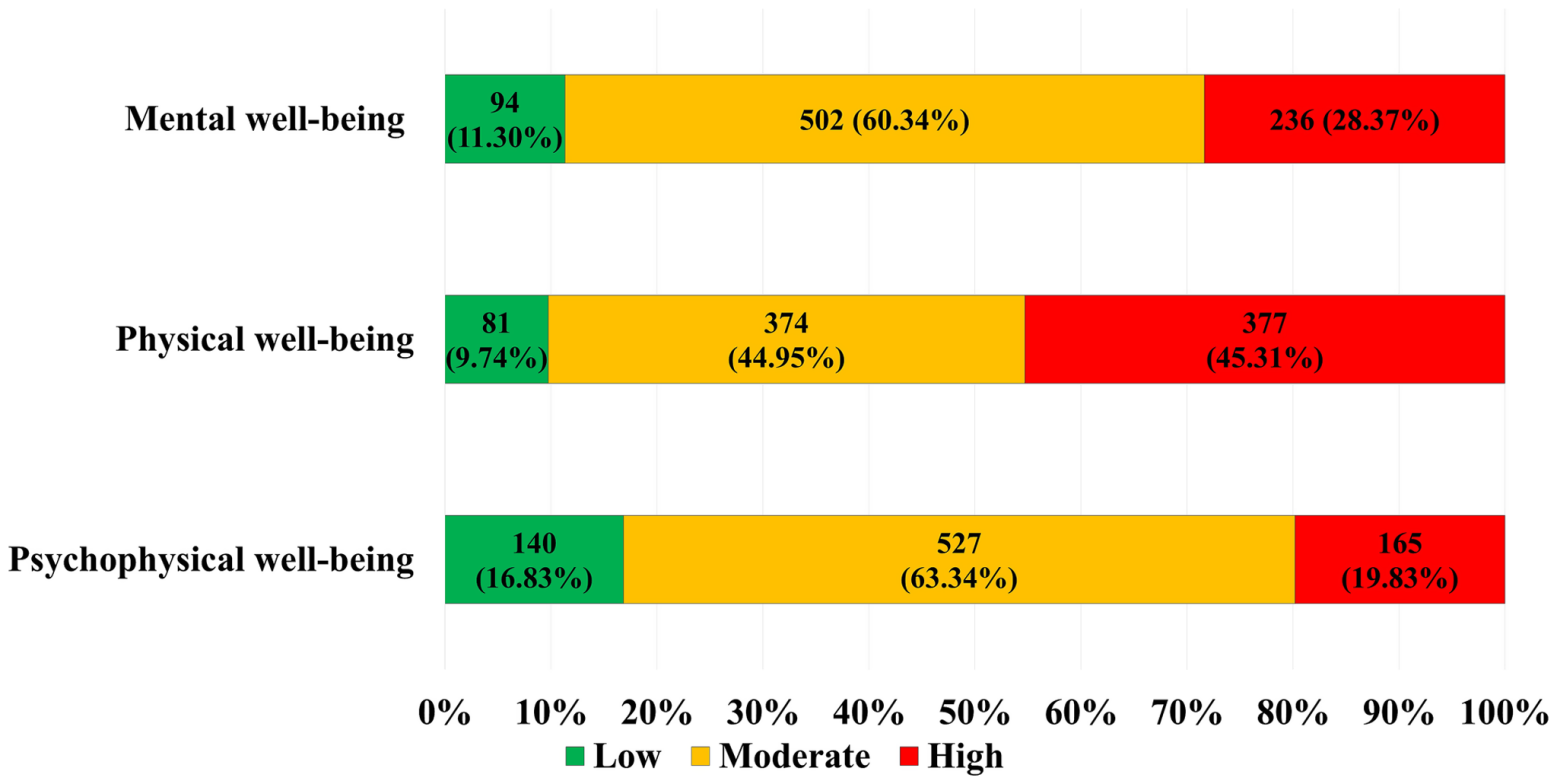
Characteristics of the study group, including assessment of psychophysical well-being.

The characteristics of the studied group of physicians, taking into account the assessment of psychophysical well-being, are presented in Figure 1.

Almost 20% (165; 19.83%) of physicians showed low values of psychophysical well-being, and as many as approx. 45% - physical well-being.

As shown in Table 2 men were characterized by significantly higher (*p* < 0.001) psychophysical well-being compared to women. Physicians working in surgical wards were characterized by significantly higher (*p* < 0.01) psychophysical well-being than those working in non-surgical wards. Older doctors were characterized by significantly higher psychophysical well-being (*p* = 0.02) than younger ones.

**Table 2:** Characteristics of the study group, taking into account the comparison of the number of points obtained in the well-being scale (D) of the Psychosocial Working Conditions Questionnaire and sociodemographic data.

Variables		Descriptive statistics							Z^1^/H^2^	*p*
		M	SD	Me	Q1	Q3	Min.	Max.		
Sex	Women	3.51	0.64	3.55	3.18	3.95	0	4.95	6.94^1^	<0.001*
	Men	3.80	0.49	3.82	3.55	4.09	0	5.00		
Age	≤30 years	3.62	0.54	3.68	3.32	4.05	0	4.45	25,658^2^	0.02*
	31–40	3.57	0.55	3.64	3.23	3.95	0	5.00		
	41–50	3.65	0.70	3.73	3.36	4.02	0	5.00		
	≥51 years	3.69	0.65	3.77	3.41	4.09	0	4.95		
*Post-hoc* analyses: 31–40: ≥51 years - *p* = 0.02										
Length of service	1–10	3.59	0.52	3.64	3.27	4.00	2.77	4.59	8,817^2^	0.06
	11–20	3.66	0.65	3.68	3.32	4.05	2.77	4.59		
	21–30	3.59	0.82	3.73	3.36	4.05	2.77	4.59		
	≥31	3.78	0.42	3.82	3.41	4.09	2.77	4.59		
Type of branch	Treatment	3.72	0.47	3.73	3.41	4.05	2.09	5.00	−2.68^1^	<0.01*
	Non-surgical	3.57	0.65	3.64	3.27	4.00	0	5.00		
Additional employment	Yes	3.62	0.61	3.68	3.32	4.02	0.00	5.00	−0.5^1^	0.60
	No	3.60	0.59	3.68	3.27	4.00	0.00	4.77		
Working on duty	Yes	3.63	0.59	3.68	3.32	4.00	0	5.00	−0.95^1^	0.343
	No	3.58	0.64	3.68	3.23	4.05	0	4.82		

M, mean; SD, standard deviation; Me, median; Q1, lower quartile; Q3, upper quartile; Z1, ^1^Mann -Whitney U test result; H2, ^2^Kruskal-Wallis test result; *p*, statistical significance; *statistically significant result.

As presented in Table 3 men were characterized by significantly higher (*p* < 0.001) physical well-being compared to women. Physicians working in non-surgical wards were characterized by significantly higher (*p* = 0.02) physical well-being than those working in surgical wards. Physicians with additional employment were characterized by significantly higher (*p* = 0.04) physical well-being than those without it.

**Table 3:** Characteristics of the study group, taking into account the comparison of the number of points obtained in the physical well-being scale (D1) of the Psychosocial Working Conditions Questionnaire and sociodemographic data.

Variables		Descriptive statistics							Z^1^/H^2^	*p*
		M	SD	Me	Q1	Q3	Min.	Max.		
Sex	Women	3.66	0.72	3.77	3.27	4.18	0	4.91	6.47^1^	<0.001*
	Men	3.97	0.54	4.00	3.64	4.36	0	5.00		
Age	≤30 years	3.85	0.63	4.00	3.55	4.36	0	4.82	4,593^2^	0.204
	31–40	3.75	0.63	3.91	3.36	4.18	0	5.00		
	41–50	3.75	0.78	3.82	3.36	4.27	0	5.00		
	≥51 years	3.77	0.71	3.91	3.55	4.18	0	5.00		
Length of service	1–10	3.80	0.60	3.91	3.45	4.27	0.00	5.00	0.182^2^	0.981
	11–20	3.77	0.73	3.91	3.36	4.27	0.00	5.00		
	21–30	3.71	0.89	3.91	3.36	4.18	0.00	5.00		
	≥31	3.80	0.50	3.86	3.50	4.18	2.73	4.73		
Type of branch	Treatment	3.73	0.73	3.91	3.36	4.18	0	5.00	−2.29^1^	0.02*
	Non-surgical	3.88	0.53	3.91	3.55	4.27	2.00	5.00		
Additional employment	Yes	3.81	0.67	3.91	3.50	4.27	0.00	5.00	−2.00^1^	0.04*
	No	3.72	0.68	3.82	3.36	4.18	0.00	5.00		
Working on duty	Yes	3.80	0.66	3.91	0	5.00	3.45	4.27	−1.81^1^	0.070
	No	3.69	0.72	3.82	0	4.82	3.27	4.18		

M, mean; SD, standard deviation; Me, median; Q1, lower quartile; Q3, upper quartile; Z1, Mann -Whitney U test result; H2, Kruskal-Wallis test result; *p*, statistical significance; *statistically significant result.

As demonstrated in Table 4 women showed significantly lower (*p* < 0.001) psychological well-being than men (*p* < 0.001). Older doctors showed significantly higher (*p* < 0.001) psychological well-being than younger ones. Physicians with longer professional experience demonstrated significantly higher (*p* < 0.001) psychological well-being than those with shorter professional experience. Physicians working in surgical wards demonstrated significantly higher (*p* < 0.01) psychological well-being than those working in non-surgical wards.

**Table 4:** Characteristics of the study group, taking into account the comparison of the number of points obtained in the mental well-being scale (D2) of the Psychosocial Working Conditions Questionnaire and sociodemographic data.

Variables		Descriptive statistics							Z^1^/H^2^	*p*
		M	SD	Me	Q1	Q3	Min.	Max.		
Sex	Women	3.35	0.66	3.45	3.00	3.82	0	5.00	6.14^1^	<0.001*
	Men	3.62	0.55	3.64	3.27	4.00	0	5.00		
Age	≤30 years	3.38	0.56	3.45	3.09	3.73	0	4.55	31,138^2^	<0.001*
	31–40	3.39	0.60	3.45	3.00	3.82	0	5.00		
	41–50	3.54	0.70	3.64	3.27	3.95	0	5.00		
	≥51 years	3.61	0.68	3.73	3.45	4.00	0	5.00		
Post-hoc analyses: ≤30 years: 41–50 - *p* < 0.01; ≤30 years: ≥51 years - *p* < 0.001; 31–40: 41–50 - *p* < 0.01; 31–40: ≥51 years - *p* < 0.001										
Length of service	1–10	3.38	0.56	3.45	3.00	3.73	0.00	5.00	36,762^2^	<0.001*
	11–20	3.54	0.65	3.55	3.18	3.91	0.00	5.00		
	21–30	3.47	0.81	3.64	3.27	3.91	0.00	5.00		
	≥31	3.76	0.48	3.91	3.45	4.09	2.55	4.64		
Post-hoc analyses: 1–10: 11–20 - *p* < 0.01; 1–10: 21–30 - *p* = 0.01; 1–10: ≥31 - *p* < 0.001; 11–20: ≥31 - *p* < 0.01										
Type of branch	Treatment	3.55	0.53	3.55	3.27	3.91	1.91	5.00	−2.71^1^	<0.01*
	Non-surgical	3.41	0.67	3.45	3.09	3.82	0	5.00		
Additional employment	Yes	3.44	0.64	3.45	3.09	3.82	0.00	5.00	1.00^1^	0.33
	No	3.49	0.60	3.55	3.09	3.82	0.00	4.73		
Working on duty	Yes	3.46	0.62	3.55	3.09	3.82	0	4.82	0.30^1^	0.762
	No	3.46	0.66	3.45	3.09	3.91	0	5.00		

M, mean; SD, standard deviation; Me, median; Q1, lower quartile; Q3, upper quartile; Z1, Mann -Whitney U test result; H2, Kruskal-Wallis test result; *p*, statistical significance; *statistically significant result.

The characteristics of the study group, including the assessment of physical activity, are presented in Table 5. Less than half of the respondents declared that they engaged in regular physical activity, and only less than 3% of them devoted 2 or more hours to a single training session per week.

**Table 5:** Characteristics of the study group of doctors, taking into account the assessment of activity physical.

Variables		Number (%)
Taking regular physical activity	Yes	376 (45.19%)
	No	456 (54.81%)
Frequency of physical activity	1–2 times	219 (26.325)
	3–4 times	147 (17.67%)
	Most days of the week	31 (3.73%)
	Every day	12 (1.44%)
	Sometimes	216 (25.96%)
	I do not exercise regularly	203 (24.40%)
Time spent on a single training session	up to 30 min	209 (25.12%)
	30–60 min	279 (33.53%)
	60–90 min	121 (14.54%)
	2 h and/or more	21 (2.52%)
	No data	202 (24.29%)
Assessment of physical activity (IPAQ)	Low	206 (24.76%)
	Moderate	266 (31.97%)
	High	360 (43.27%)

According to Table 6 the surveyed physicians spent the most time on low-intensity physical activity (1488.05 MET-minutes/week).

**Table 6:** Characteristics of the study group including descriptive statistics regarding the duration of physical activity expressed in MET-minutes/week depending on its intensity level.

Variables	Low	Moderate	High	Total value
Mean	1488.05	501.84	853.41	2,821,184
Standard deviation	1415.72	751.69	1186.08	2,301,872
Median	990.00	240.00	480.00	2251.75
Lower quartile	297.00	0.00	0.00	1035.00
Upper quartile	2772.00	720.00	1440.00	4178.00
Minimum	0.00	0.00	0.00	0.00
Maximum	4158.00	5040.00	7200.00	10000.00
Statistical significance of the Shapiro -Wilk test	<0.001	<0.001	<0.001	<0.001

As shown in Table 7 there was significant difference in physical activity between female and male doctors (*p* < 0.001) — more women with low physical activity. Moreover, doctors on duty more often showed a high level of physical activity (*p* = 0.01; 303, 45.91 vs. 57, 33.14%).

**Table 7:** Characteristics of the study group, taking into account the analysis of differences in physical activity, gender, age, having children and the number of children, as well as taking up on-call work, duration of on-call work, taking up additional work and length of service.

Variables		Low physical activity (206; 100%)	Moderate physical activity (266; 100%)	High physical activity (360; 43.27%)	Chi^2^	*p*
Sex	Women	115 (28.26%)	190 (23.60%)	155 (43.06%)	16,984	<0.001*
	Men	91 (22.55%)	76 (37.25%)	205 (40.20%)		
Age [years]	25–30	37 (18.05%)	78 (38.05%)	90 (43.90%)	14,488	0.02*
	31–40	73 (23.40%)	95 (30.45%)	144 (46.15%)		
	41–50	49 (27.84%)	56 (31.82%)	71 (40.34%)		
	>50	47 (27.84%)	37 (30.45%)	55 (46.15%)		
Having offspring	Yes	140 (27.13%)	159 (30.81%)	217 (42.05%)	4,120	NS
	No	66 (20.89%)	107 (33.86%)	143 (45.25%)		
Number of children	0	84 (29.47%)	75 (26.31%)	126 (44.21%)	10,184	0.04*
	1–2	93 (22.96%)	142 (35.06%)	170 (41.98%)		
	≥3	13 (19.40%)	28 (41.79%)	26 (38.81%)		
Specialization	WITH	57 (20.80%)	84 (30.66%)	133 (48.54%)	5,427	NS
	NZ	149 (26.70%)	182 (32.62%)	227 (40.68%)		
On duty	Yes	155 (23.48%)	202 (30.61%)	303 (45.91%)	9,098	0.01*
	No	51 (29.65%)	64 (37.21%)	57 (33.14%)		
Average hours of work on duty	0	51 (31.10%)	60 (36.59%)	53 (32.32%)	17,267	0.03*
	≤12	23 (23.96%)	36 (37.50%)	37 (38.54%)		
	13–24	100 (23.26%)	126 (29.30%)	204 (47.44%)		
	25–48	30 (25.00%)	38 (31.67%)	52 (43.33%)		
	>48	2 (9.09%)	6 (27.27%)	14 (63.64%)		
Additional employment	Yes	141 (24.48%)	185 (32.12%)	250 (43.40%)	0.079	NS
	No	65 (25.39%)	81 (31.64%)	110 (42.97%)		
Length of service [years]	1–10	100 (21.83%)	156 (34.0%)	202 (44.10%)	10,737	NS
	11–20	51 (28.49%)	56 (31.28%)	72 (40.22%)		
	21–30	34 (25.95%)	32 (24.43%)	65 (49.62%)		
	≥31	21 (32.81%)	22 (34.38%)	21 (32.81%)		

Z, surgical specialization; NZ, non-surgical specialization; Chi ^2^, ^Chi 2^ statistic value; p, statistical significance; *statistically significant result; NS, no significance.

The highest level of physical activity was also observed among doctors aged 31–40 (*p* = 0.02), having 1–2 children (*p* = 0.04) and working an average of 13–24 h on duty (*p* = 0.03).

As presented in Table 8 Spearman rank correlation analysis did not reveal any significant correlations between psychophysical well-being and physical activity of the surveyed Polish doctors.

**Table 8:** Results of the correlation analysis between the number of points obtained in the individual domains of the Psychosocial Working Conditions Questionnaire and physical activity expressed in MET minutes/week.

Variables	*R*	*p*
Total points & MET-min/week	0.051	0.145
Physical well-being scale points & MET-min./week	0.064	0.066
Points on the scale of mental well-being & MET-min./week	0.033	0.346

R, Spearman’s rank correlation coefficient; *p*, statistical significance.

## Discussion

In our own study, out of the entire group (832; 100%), almost 20% (165; 19.83%) of doctors showed a low value of psychophysical well-being. Moreover, as many as approx. 45% described their physical well-being as low, and their psychological well-being – approx. 30%. The values shown in our own study are relatively high. According to the definition given in the introduction to the presented work, well-being can be identified with quality of life. M. Walkiewicz et al. in their work on the quality of life of doctors-graduates of the Medical University of Gdańsk showed different results. In the aforementioned study covering 255 doctors, the respondents declared a good quality of life, exceeding the general quality of life of Poles and people in a similar age group (25–34) according to the Social Diagnosis 2011 report (24, 25).

Our study also showed significantly lower values of all three parameters studied (psychophysical, mental and physical well-being) in women compared to men. According to the literature, women are more critical of themselves and their lives and assess their quality worse. There are studies on the quality of life and mental well-being in many groups, including, e.g., cardiology patients (26), premenopausal women (27), or young adults (28), confirming lower life satisfaction in women compared to men. However, there is a lack of such studies among medical personnel, including physicians.

Taking into account other variables studied, it was shown that the assessment of doctors’ well-being was influenced by, among others: seniority, age, or type of work performed. The worst psychophysical well-being was demonstrated by people aged 31 to 40, and the worst psychological well-being was even among younger people. This is probably due to the fact that the mentioned period in a doctor’s life is most often the completion of one and perhaps the beginning of another specialization, and additionally seeing patients in clinics and working on duty. Moreover, in the present times it is the period of starting a family and raising children, which is associated with additional responsibilities. This is a difficult period in the life of every young adult, and especially among doctors who devote a lot of time to learning and building their professional career, often combining it with their private life, which is quite a challenge. The group discussed also includes people who devote themselves to a medical career and give up having a family at that time. They may not feel fully fulfilled in this respect. On the other hand, young female doctors raising children at this time, so they can be excluded from the labor market and unable to build their professional career. They often feel unfulfilled, especially if, for example, their husband-doctor is successful in his profession at that time. This is also the time when decisions are made about loans or buying apartments, which is associated with financial losses for young doctors, whose earnings are often not fully satisfactory. Older doctors are already relatively settled, have a strong position in the workplace, raised children and their regular patients, which is why their assessment of well-being may be better, compared to young ones. A significant difference was also shown in the type of ward in which the surveyed doctors worked: better psychophysical and mental well-being was demonstrated by those working in surgical wards, and physical well-being – by those working in non-surgical wards. This can be interpreted in such a way that work in surgical wards gives doctors greater satisfaction, but is more physically demanding. In addition, additional employment had a positive effect on better physical well-being, and longer work experience – on mental well-being. Other researchers have also shown differences in the mental and physical workload of doctors depending on the type, amount and duration of work performed, but there are very few such studies in the literature (29).

In our other studies, using the same questionnaire to assess psychophysical well-being as in the presented study, but in the group of 1,080 (100%) nurses, more than half of them (735; 68%) showed average psychophysical well-being, and 179 (16.6%) — low. Widows and widowers were characterized by lower psychophysical well-being than married or single nurses. Interestingly, nurses who took on additional work had better well-being than those who did not work additionally (30).

In our own study, less than half of the respondents (376; 45.19%) declared that they were taking part in regular physical activity, and only about 3% (21; 2.52%) of the respondents devoted 2 or more hours to a single training session per week. Comparing this to the WHO recommendations cited in the introduction, this was definitely too little (14). According to research conducted by the Ministry of Tourism and Sport, only 28% of adult Poles followed the above recommendations (31). In the analyzed group of doctors using the IPAQ questionnaire, high physical activity was found in a larger group, i.e., in over 40% (360; 43.27%) of the examined. In the analysis conducted by M. Gacek in the group of doctors, their level of participation in physical activity was low — they most often declared undertaking physical activity once a week (32). J. Korpak-Baj et al. observed, however, that only 15% of doctors in their study showed a low level of physical activity, with female doctors being more active (33). S. Calongue-Pascual et al. showed that about half of the doctors analyzed by them considered themselves physically active, claiming that it helps them in recommending physical activity to their patients (34).

In the world literature, one can find many studies on the physical activity of other medical personnel, most often nurses or medical students. However, there are very few studies on doctors.

For example, P. Tuominen conducted an analysis of the physical activity of teachers, nursing staff and ICT workers. The study did not show a significant difference between nursing staff and other professional groups in terms of the declared intensity and frequency of physical activity, although the average intensity of nurses and ICT workers was slightly higher than among teachers (35). The level of physical activity of nurses examined by S. Chappel et al. largely consisted of low-intensity physical activity interspersed with medium-intensity activity (36). F. Roskoden et al. showed a significant difference in the scope of physical activity between shift workers and non-shift workers (*p* < 0.01) and between shift-worker nurses (median = 2.1 METs SE = 0.1) and non-shift-worker administrative staff (median = 1.5 METs SE = 0.07, *p* < 0.05). In the cited study, shift work did not affect the overall physical activity of the study participants (37). In our study, more women than men were characterized by low physical activity. Moreover, people working on duty more often showed a high level of physical activity than those who did not do this type of work (303; 45.91 vs. 57; 33.14%).

In addition, H. Blake et al. examined the level of physical activity of nursing and medical students. They showed that many students did not reach the recommended minimum level of physical activity (nursing – 48%, medical – 38%), and the most noticeable barriers to exercising were: lack of time and inconvenient and tight schedule of studies or internships (38). H. Alzahtani et al. examined medical students from Saudi Arabia and showed that their level of physical activity was low. They also indicated insufficient knowledge of the guidelines for physical activity among the participants of their study (39). In our other studies on nursing staff, we showed that 848 (80.5%) reached a sufficient level of physical activity (>600 METs), and the remaining 206 (19.5%) — too low (below 600 METs) (40).

As suggested by H. Fibbins et al., perhaps counseling on physical exercise should be introduced for medical personnel. They showed that cardiorespiratory fitness of medical personnel they studied significantly improved after education on physical activity (*p* < 0.001), and significant improvements occurred with a decrease in the time spent in a sedentary position (*p* < 0.0005) and an increase in moderate to vigorous physical activity (*p* < 0.005) (41). A systematic review of 18 studies involving 11,500 medical students from 13 countries found that physical activity was negatively associated with burnout and positively associated with quality of life. Higher exercise intensity and frequency led to greater benefits in both areas. Its authors suggest that incorporating physical activity into curricula can improve student well-being and better prepare them for the demands of medical work (42). Another study of 1,060 residents and fellows at the Mayo Clinic in Rochester, Minnesota, assessed the impact of a 12-week, team-based, incentive-based exercise program on physical activity levels, quality of life, and burnout. Results showed that participants were more likely to meet physical activity recommendations (48% vs. 23%), had higher quality of life (median 75 vs. 68), and lower levels of burnout (24% vs. 29%) than nonparticipants (43).

There are many scientific studies proving the beneficial effect of physical activity on psychophysical well-being, quality of life and happiness (44–48). For example, a study of 4,520 physicians working in Chinese psychiatric hospitals found that higher frequency of physical activity was associated with lower levels of depression and anxiety and higher levels of happiness (49). Our study analysed the relationship between the IPAQ questionnaire result with individual domains of the psychophysical well-being questionnaire and no significant relationship was demonstrated. Similarly, our other studies conducted in a group of nurses, did not show such relationships (50). Working conditions, occupational stress, sleep quality, social relationships, and mental health are among the numerous factors that may weaken or obscure the potential impact of physical activity on psychophysical well-being. In professional groups such as doctors and nurses, the beneficial effect of physical exercise on well-being may also be diminished by chronic fatigue, work overload, and high stress levels. Additionally, it is feasible that the absence of beneficial effects on well-being may be attributable to the fact that physical activity in this group was predominantly mandatory (work-related) rather than recreational.

Studies conducted in 2016–2017 by the Medical self-government in Poland in a group of two and a half thousand doctors and dentists suggested that doctors worked on average (excluding shifts) 165.1 h per month, and dentists – 147.5 h per month. Total working time (including shifts) amounted to an average of 234.3 and 156.8 h per month, respectively. Specialized doctors worked even more. The report shows that specialists in palliative medicine (338.9 h per month), emergency medicine (336.1 h per month) and anesthesiology and intensive care (301.6 h per month) worked the most. The average number of jobs was also 3. In the professional group of doctors, men worked almost 36 h more per month than women (255 vs. 219.1, respectively), and among dentists – over 22 h (173.7 vs. 151.3, respectively) (51). With such a busy and demanding work schedule, it is difficult to maintain the correct principles of a healthy lifestyle, including regular physical activity.

In conclusion, the topic presented in the paper is important to investigate, because many studies in the literature on the studied topic focus on other medical personnel, mainly nurses, while there is a lack of similar studies on doctors. It may be worth expanding the group and examining also other elements of doctors’ lifestyle and their risk behaviors, as well as their consequences.

The presented study has several significant limitations that should be taken into account when interpreting the results. The methodology used self-report tools, such as the International Physical Activity Questionnaire (IPAQ) and the theoretical D scale, increases the risk of cognitive errors and response bias, including the social desirability effect. Potential confounding factors, such as sleep quality, the presence of chronic diseases, the level of occupational stress or family conditions, have also not been ruled out, which may also affect the results. Additionally, the short version of the IPAQ questionnaire does not allow for distinguishing between recreational and professional physical activity.

Despite the indicated limitations, the results of the study have practical significance and can be the basis for planning preventive and interventional activities in the medical environment. The conclusions indicating unsatisfactory psychophysical well-being of doctors and their average level of physical activity can be used to design health support programs and improve the working conditions of medical personnel. Additionally, the study can be a starting point for further, in-depth analyses covering a wider range of psychological and environmental variables.

## Conclusion

The psychophysical well-being of Polish doctors was unsatisfactory, worse in women, young adults and those with shorter work experience and those working in non-surgical departments and was not related to the physical activity of the respondents in any way. The physical activity of the Polish doctors studied, assessed by International Physical Activity Questionnaire, was average, and female doctors and non-on-duty people were characterized by its lower level. It was also not related to and did not affect the psychophysical well-being of the surveyed doctors. The habits of physical activity and their working conditions of Polish doctors require improvement. Educational and support programs should be introduced aimed at improving the quality of work and health behaviors of Polish doctors.

## Data Availability

The datasets presented in this article are not readily available because all data used during the current study is the property of the authors and are available from the corresponding author on reasonable request. Requests to access the datasets should be directed to jdabek@sum.edu.pl.
